# Self-sacrifice Template Formation of Hollow Hetero-Ni_7_S_6_/Co_3_S_4_ Nanoboxes with Intriguing Pseudo-capacitance for High-performance Electrochemical Capacitors

**DOI:** 10.1038/srep20973

**Published:** 2016-02-11

**Authors:** Hui Hua, Sijia Liu, Zhiyi Chen, Ruiqi Bao, Yaoyao Shi, Linrui Hou, Gang Pang, Kwun Nam Hui, Xiaogang Zhang, Changzhou Yuan

**Affiliations:** 1 School of Materials Science & Engineering, Anhui University of Technology, Maanshan, 243002, P.R. China; 2 Institute of Applied Physics and Materials Engineering, Faculty of Science and Technology, University of Macau, Macau, P.R. China.; 3 College of Material Science & Engineering, Nanjing University of Aeronautics and Astronautics, Nanjing, 210016, P.R. China

## Abstract

Herein, we report a simple yet efficient self-sacrifice template protocol to smartly fabricate hollow hetero-Ni_7_S_6_/Co_3_S_4_ nanoboxes (Ni-Co-S NBs). Uniform nickel cobalt carbonate nanocubes are first synthesized as the precursor *via* solvothermal strategy, and subsequently chemically sulfidized into hollow heter-Ni-Co-S NBs through anion-exchange process. When evaluated as electrode for electrochemical capacitors (ECs), the resultant hetero-Ni-Co-S NBs visually exhibit attractive pesudo-capacitance in KOH just after continuously cyclic voltammetry (CV) scanning for 100 cycles. New insights into the underlying energy-storage mechanism of the hollow hetero-Ni-Co-S electrode, based on physicochemical characterizations and electrochemical evaluation, are first put forward that the electrochemically induced phase transformation gradually occurrs during CV sweep from the hetero-Ni-Co-S to bi-component-active NiOOH and CoOOH, which are the intrinsic charge-storage phases for the appealing Faradaic capacitance (~677 F g^−1^ at 4 A g^−1^) of hollow Ni-Co-S NBs at high rates after cycling. When further coupled with negative activated carbon (AC), the AC//hetero-Ni-Co-S asymmetric device with extended electrochemical window of 1.5 V demonstrates high specific energy density of ~31 Wh kg^−1^. Of significance, we strongly envision that hollow design concept and new findings here hold great promise for enriching synthetic methodologies, and electrochemistry of complex metal sulfides for next-generation ECs.

In recent years, electrochemical capacitors (ECs) are emerging as promising energy-storage devices with numerous appealing electrochemical merits such as long-life span, fast charge/discharge rates, and safe operational mode, and so on. And they have attracted tremendous attentions for potential applications in electric vehicles (EVs) and hybrid EVs in combination with rechargable batteries and/or fuel cells^1^,^2^. Unfortunately, ECs still suffer seriously from relatively lower specific energy density (SED) when compared to secondary batteries, as established well^2^. In the past decades, tremendous research progresses therefore have been accomplished greatly for the huge enhancement of supercapacitive properties by smartly exploring low-cost Faradaic pseudo-capacitive electrode candidates with large specific capacitances (SCs), thanks to their inherent redox-reaction-enriched charge storage mechanism^3^,^4^,^5^,^6^.

Since the pioneering contribution of SnS as an electrode for ECs by Jayalakshmi, *et al*. in 2004^7^, various transition metal sulfides (TMSs), such as amorphous CoS^8^, CoS_1.097_^9^, Co_9_S_8_^10^,^11^,^12^, Ni_3_S_2_^11^,^13^, NiS^14^, CoS_x_^15^,^16^, Co_3_S_4_^17^, CoS_2_^18^, CoS^19^, *etc.*, have been greatly stimulated, and investigated substantially as electroactive materials for ECs applications, benefiting from rich valences, desirable chemical stability and superior electrochemical performance^8^,^9^,^10^,^11^,^12^,^13^,^14^,^15^,^16^,^17^,^18^,^19^. In general, the pseudo-capacitive abilities of these sulfides in alkaline solutions are phenomenologically ascribed to the Faradic reacion related to the mutual transformation of the M species (M = Co, Ni) with various valences *i.e.*, M-S/M-S-OH/M-S-O, as retrieved previously^8^,^9^,^10^,^11^,^12^,^13^,^18^,^19^. Nevertheless, some direct and enough evidence is still neccessary to fully support the above statement, to the best of our knowledge. It is thus of significant importance to figure out underlying intrinsic energy storage mechanisms of TMSs in alkaline electrolytes, and thorough investigations are urgently needed to carry out.

In addition, to further improve electrochemical properties of these TMSs, one striking concept is the smart hybridization of bi-component TMSs, motivated by the rule of “1 + 1 > 2”, where even better electrochemical performance can be reasonablly anticipated *via* the synergistic effec of each constituent^20^,^21^,^22^, compared to any single-phase counterpart. Attractively, the existence of hetero-junctions at the nanoscale between the two TMSs would render an enhenced inner electroc field at their interfaces meanwhile, improving the electron transfer over the whole electrochemical reactions^21^,^22^. Furthermore, it is well established that electrochemical behaviors of pseudo-capacitors are determined significantly by the kinetic features, which are controlled by the ion transportation into the electrode materials with rich electroactive sites^19^. However, it is still greatly challengable to explore and develop facile yet effective approaches to constructing hollow/mesoporous hetero-nanoarchitectures with homogenerous interface/chemical distribution at the nanoscale.

## Results

**Figure 1:**
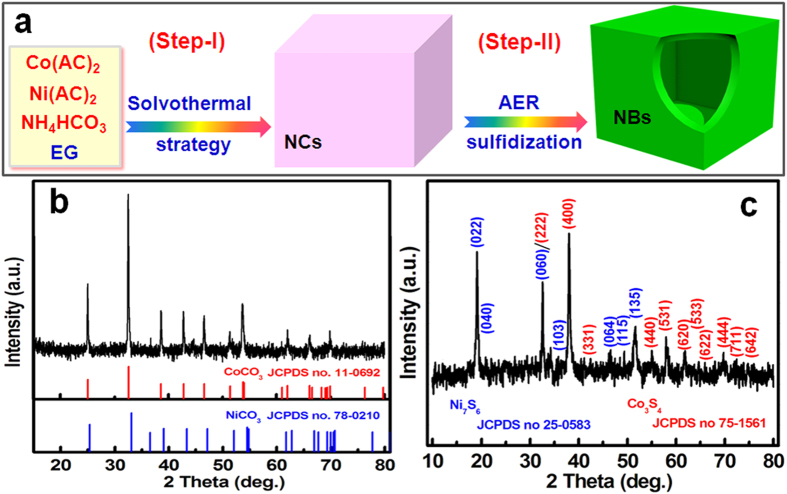
Schematic illustration of the self-sacrifice template synthetic process, (**a**) typical wide-angle XRD patterns of the as-obtained NixCoyCO3 (**b**) and hetero-Ni-Co-S NBs (**c**) products.

With these considerations mentioned above in mind, in the contribution, we sucessfully fabricated the precursor of solid-solution nickel cobalt carbonate nanocubes (denoted as Ni_x_Co_y_CO_3_ NCs) by solvothermal approach, then designed an efficient self-sacrifice template strategy to construct hollow hetero-Ni_7_S_6_/Co_3_S_4_ nanoboxes (designed as Ni-Co-S NBs) *via* shape-preserved anion exchange reaction (AER). Typical synthetic process of hollow hetero-Ni-Co-S NBs can be briefly depicted in Fig. 1a. Firstly, a scalable solvothermal approach was developed to prepare carbonate intermediate (step I), where the reaction of Co(II) and Ni(II) with CO_3_^2−^ from the decomposition of NH_4_HCO_3_ in ethylene glycol (EG) system at 200 °C yielded large-scale Ni_x_Co_y_CO_3_ NCs. As schematically demonstrated in Fig. 1a, an efficient self-sacrifice template strategy was then applied to realize the gradual carbonate-to-sulfide transformation *via* the low-cost solution-based AER (step II), where the cheap Na_2_S was utilized as a sulfidizing agent. Finally, high-quality hollow hetero-Ni-Co-S NBs were finely prepared with topotactical relationship in structure and morphology. Figure 1b shows the representative powder X-ray-diffraction (XRD) pattern of the as-obtained light pink Ni_x_Co_y_CO_3_ precursor. All the diffraction peaks can be successfully indexed to a solid-solution phase of NiCO_3_ (JCPDS card, #78-0210) and CoCO_3_ (JCPDS card, #11-0692), which should be rationally ascribed to their same Rhombohedral structure (R-3c (167)) and close lattice constants (4.6117 × 14.735 for NiCO_3_; 4.659 × 14.957 for CoCO_3_). The molar ratio of the CoCO_3_ to NiCO_3_ in this intermediate is ~14.3:5 according to the X-Ray fluorescene (XRF) data. Typical crystallographic structure of the black product after sulfidation for 4 h are examined by powder XRD measurement, as observed in Fig. 1c. It is evident that all of these diffraction peaks can be unambiguously assigned to a mixture of orthorhombic Ni_7_S_6_ with Bmmb (63) (JCPDS card, #25-0583) and Co_3_S_4_ with a cubic structure (Fd-3 m (227), JCPDS card, #75-1561). The atomic ratio of Co to Ni is ~27.5/15.1 according to the X-ray photoelectron spectra (XPS) data, the molar ratio of the Ni_7_S_6_/Co_3_S_4_ in hetero-Ni-Co-S is calculated as ~6:25 in the mixture accordingly, considering their stoichiometric ratio. All the reactions involved here can be preliminarily summarized, and expressed as follows:


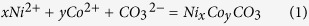



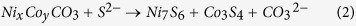


**Figure 2:**
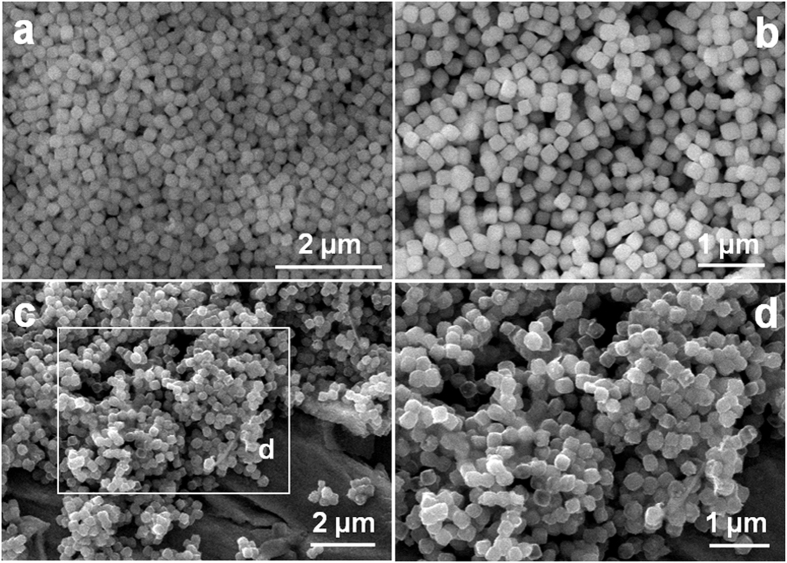
FESEM images of the as-obtained NixCoyCO_3_ (**a, b**) and hetero-Ni-Co-S NBs (_c, d_) products.

Figure 2a,b demonstrate the panoramic field-emission scanning electron microscopy (FESEM) images of the as-synthesized Ni_x_Co_y_CO_3_ precursor by various magnifications. Numerous uniform cube-shaped samples with a size of ~200 nm are observed clearly. Low-resolution FESEM images of the Ni_x_Co_y_CO_3_-derived hetero-Ni-Co-S NBs are displayed in Fig. 2c,d. Evidently, the as-obtained hetero-Ni-Co-S specimen inherits well original cube-like structure of the intermediate Ni_x_Co_y_CO_3_ without noticable size alterations. Careful examination (Fig. 2d) reveals that the resultant hetero-Ni-Co-S sample apparently possesses interior cavities, as identified from a few of NCs with several broken parts, which suggests the hollow nature of hetero-Ni-Co-S NBs product.

**Figure 3:**
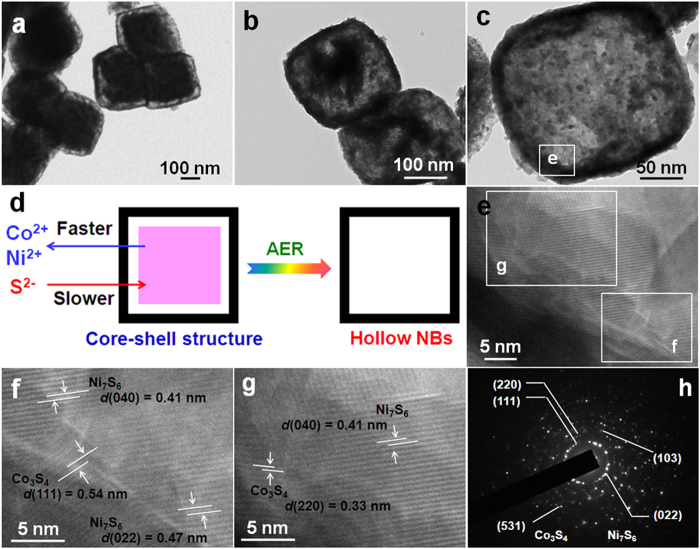
TEM images of the Ni-Co-S-0.5 (**a**) and Ni-Co-S-3 (**b**), and hollow hetero-Ni-Co-S NBs (**c**). Schematic illustration for the transformation from core-shell structure to hollow NBs (**d**), HETEM images (**e-g**) and corresponding SAED pattern (**h**) of the as-fabricated hetero-Ni-Co-S NBs.

Some parallel experiments were further carried out with different solution-based sulfidation durations to investigate the intrisic formation of hollow heter-Ni-Co-S NBs, and transmission electron microscopy (TEM) technique was carried out to monitor the structural evolution as a function of sulfidation time. Obviously, the Ni_x_Co_y_CO_3_ precursor is composed of uniform solid NCs without visible hollow interior (see Supporting Information, Fig. S1). Interestingly, it is worthy of noting that there is a visual gap of ~10 nm between well-defined shells and solid cores (Fig. 3a), rendering the formation of a unique core-shell nano-architecture (desinged as Ni-Co-S-0.5), just after sulfidation treatment for 0.5 h at 120 °C. The color change of the obtained sample from initial light pink to light black strongly confirms the partial formation of sulfides. Strikingly, the dense core turns out to even smaller and more inner voids appear for the Ni-Co-S-3 sample when further increasing the reaction time up to 3 h, as seen in Fig. 3b. Of particular note, with the sulfidation duration prolonging to 4 h, hollow box-shape structure with a single shell of ~10 nm in thickness and a completely void interior is sucessfully obtained (Fig. 3c). These findings above suggest a gradual phase conversion over the whole sulfidation process from solid Ni_x_Co_y_CO_3_ NCs to hollow hetero-Ni-Co-S NBs. The sulfidation process can be essentially described as an AER of the Ni_x_Co_y_CO_3_ NCs with the anion of S^2−^ (see the eq. 2), where the discrepancy in diffusion rate between metal cations (*i.e.*, Ni^2+^ and Co^2+^) and sulfide anion (S^2−^) take places^19^,^23^. The AER occurrs in a way that the outward diffusion of Ni^2+^/Co^2+^ is even faster than the inward diffusion of S^2−^ ions, thus creating inner voids progressively with AER proceeding, and finally forming hollow Ni-Co-S NBs on the completion of the AER, as shown in Fig. 3d. A high-resolution TEM (HRTEM) image (Fig. 3e), discerned from a sampling area indicated by the white rectangle in Fig. 3d, presents clear lattice fringes in various orientations, suggesting the crystalline nature of the Ni-Co-S NBs. Furthermore, HRTEM visualizations (Fig. 3f,h) display clear lattice fringes with various interplanar distance in different regions. As observed in Fig. 3f, taken from the white rectangle region as indicated in Fig. 3e, well-defined lattice finges with the spacing are ~0.41 and ~0.54 nm, which can be attributed to the (040) crystalline plane of the Ni_7_S_6_ and (111) facet for the Co_3_S_4_, respectively. Similar phenomenon also can be observed in Fig. 3g, which is corresponding to the magnified white square zero in Fig. 3e. The observations above cogently confirm that desirable hetero-junctions are well dipersed at the nanoscale between two nano-phases of Ni_7_S_6_ and Co_3_S_4_, which benefits from the inherent solid-solution nature of Ni_x_Co_y_CO_3_ with homogeneity at an atomic level, as described by the above XRD analysis (Fig. 1b). The selected area electron diffraction (SAED) pattern, as seen from Fig. 3h, illustrates a series of concentric rings, revealling the polycrystalline characteristics of the hetero-Ni-Co-S NBs, and matches well with (220), (111) and (531) crystalline planes of the Co_3_S_4_, and (103) and (022) facets of the Ni_7_S_6_, respectively, which are in good aggreement with the aformentioned XRD data (Fig. 1c).

## Discussion

**Figure 4:**
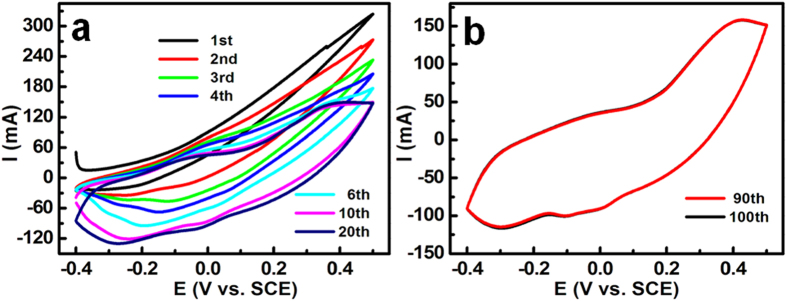
CV curves (20 mV s^–1^) of the hetero-Ni-Co-S NBs electrode with different cycles as indicated.

In view of these intriguing structural and compositional advantages described above, the unique hollow hetero-Ni-Co-S NBs guarantee large electrode/electrolyte contacting sur-/interfaces, short and convenient ionic diffusion, bi-component-active Co_3_S_4_ and Ni_7_S_6_, and desirable electronic transportation, which would be greatly favorable for enormous enhancement in electrochemical properties as an electrode for advanced ECs. Electrochemical performance of the Ni-Co-S NBs is exmained first by cyclic voltammetry (CV) test in a standard three-electrode configuration using 6 M KOH as electrolyte. The CV test is conducted in a potential interval between –0.4 and 0.5 V (*vs.* SCE), as demonstrated in Fig. 4a,b. Figure 4a collects the typical CV curves of the initial 20 cycles recorded at a scanning rate of 20 mV s^−1^ for the hetero-Ni-Co-S NBs electrode. Remarkably, the CV image is distinct from each other with the respect to cycle number, as evident in the *E*-*I* responses (Fig. 4a), indicating different electrochemical processes involved in these CV cycles. As regards to the first sweep cycle, both of the electrochemically cathodic and anodic current waves are nearly located above the zero-current baseline, which strongly authenticates the inherently poor supercapacitive behaviors of the hetero-Ni-Co-S electrode itself in aqueous KOH electrolyte. Obviously, anodic current responses turn out to be less and less, while the cathodic ones become larger and larger concomitantly with further CV sweep. The gradually increasing area integrated below zero-current line with cycling signifies the promotion of the available charges stored in the electrode with the CV scanning. Furthermore, the positive sweep exhibits more mirror-image symmetric to its counterpart on the negative scanning, as the cycle number is up to the 20^th^ cycle. It is therefore easy to conclude that the electrode presents much better electrochemical capacitance after 20 cycles, compared to that of the 1^st^ one. Different CV shapes between the two additionally manifest their distinct charge-storage processes. In a contrast, the CV shape impressively changes little between the 20^th^ and 100^th^ cycles, as shown in Fig. 4a,b, which suggests the same electrochemical reaction occurring over these CV scanning cycles. Furthermore, almost no any differences are discernable in either the integrated area or shape of the voltammograms between the 90^th^ and 100^th^ cycles. It is thus affirmative that the electrochemical response is extraordinarily stable after uninterrupted 90 cycles. Based on the aforementioned discussions, the appealing electrochemical performance in 6 M KOH observed for the hetero-Ni-Co-S NBs after the 100^th^ cycle should be tentatively attributed to the fascinating contribution from newly-formed electoactive phases, which are electrochemically formed during continuously cycling, rather than the fresh hetero-Ni-Co-S electrode itself, that is, electrochemical phase-transformation process must progressively takes place with cycling in 6 M KOH electrolyte.

**Figure 5:**
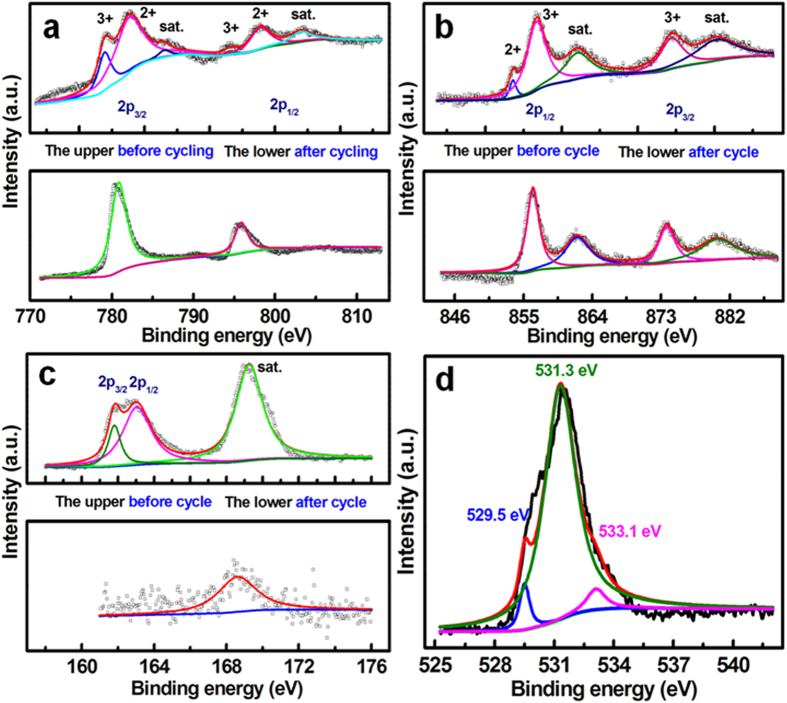
XPS survey spectra and fitting data of the as-obtained hetero-Ni-Co-S NBs electrode after and before the 100th cycle as indicated: Co 2p (**a**), Ni 2p (**b**) and S 2p (**c**). The O 1s (**d**) for the electrode after cycling for 100 times.

**Figure 6:**
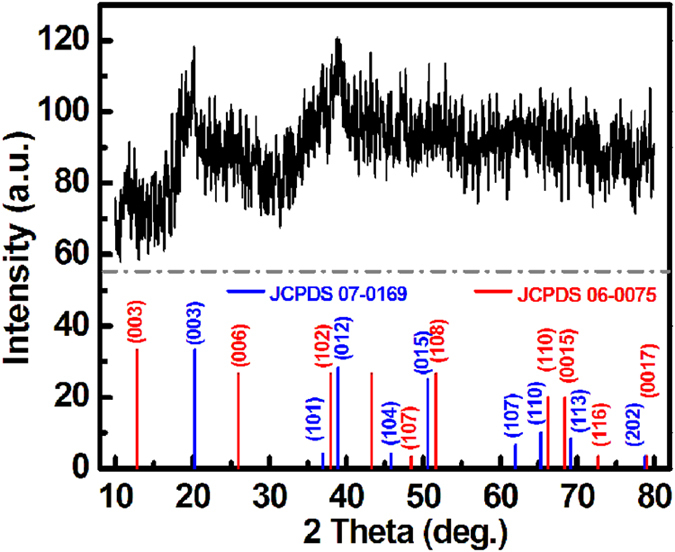
XRD pattern of the hollow hetero-Ni-Co-S NBs electrode after the 100th CV cycle.

To further clarify the underlying electrochemical conversion with CV cycling, XPS measurements were performed accordingly, and representative XPS spectra before and after the 100^th^ cyle are illustrated in Fig. 5. The Ni 2p (the upper in Fig. 5a) and Co 2p (the upper in Fig. 5b) high-resolution XPS spectra of the fresh Ni-Co-S NBs are carefully fitted, and analyzed considering two spin-orbit doublets. Then, we can draw a conclusion that the fresh Ni-Co-S NBs contains Ni(III), Ni(II), Co(III) and Co(II), where the atomic ratios of the Co^3+^/Co^2+^ and Ni^3+^/Ni^2+^ are calculated as ~5/18 and ~2/1, respectively. Strikingly, the sample possesses only two kinds of cations including Ni^3+^ and Co^3+^ after cycling, as observed from the lower panels in Fig. 5a,b, that is, all divalent cations are wholly transfomed into trivalent ones during the contineous CV sweep. The fitting data of the S 2p XPS spectra of the hetero-Ni-Co-S NBs before and after cycling are fitted and summarized in Fig. 5c. The peaks located at binding energies (BEs) of ~161.8 and ~163.0 eV belong to S 2p_3/2_ and S 2p_1/2_, while the peak at BE of ~169.3 eV can be assigned to the shake-up satellite (sat.) structure^24^. Note that the S content in the Ni-Co-S NBs before CV cycle is ~57.4 at.%, which is much larger than that (just around 0.72 at.%) after the 100^th^ cycle. In contrast, the content of O species unexpectedly increases up to ~70 at.% after CV scanning for 100 cycles. High-resolution O 1 s spectra are further depicted in Fig. 5d, in which a broad asymmetric curve is observed apparently, and de-convoluted into three peaks at BEs of ~529.5, ~531.3 and ~533.1 eV, respectively, corresponding to the typical bands of oxygen in oxides (Metal-O-Metal, O1), hydroxides (metal-OH, O2) and bound water (O3), repspectively^25^. Evidently, the O1 and O2 (*i.e*., oxy-hydroxide) is dominant (~98 at.%) in oxygen species from the product obtained after cycling. Figure 6 demonstrates the typical XRD pattern of the hetero-Ni-Co-S electrode after the 100^th^ cycle. Clearly, a mixture of rhpmbohedral CoOOH (JCPDS card, #07-0169, R-3 m (166)) and hexagonal phase NiOOH (JCPDS card, #06-0075) can be found clearly after cycling. As analysized above, the whole electrochemical process during cycling can be expressed that the phase transformations from Ni_7_S_6_ (or Co_3_S_4_) to Ni(OH)_2_ (or Co(OH)_2_) irreversibly occurs first, followd by electrochemical oxidation from Ni(OH)_2_/Co(OH)_2_ to Ni(III)OOH/Co(III)OOH^26^. Then, reversible electrochemical reactions take palce in the subsequent CV cycle measurements. As a result, the newly-formed Ni(III)OOH and Co(III)OOH should rationally elucidate the appealing electrochemical capacitance observed after the 100^th^ cycle. The electrochemical mechanism over cycling in 6 M KOH for the hetero-Ni-Co-S can be summarized, and the phase-transformation reactions involved here are rationally proposed as follows:


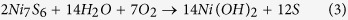



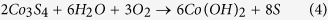


The electrochemically reversible redox reactions can be expressed by following equations:


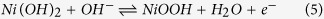



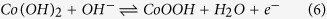


**Figure 7:**
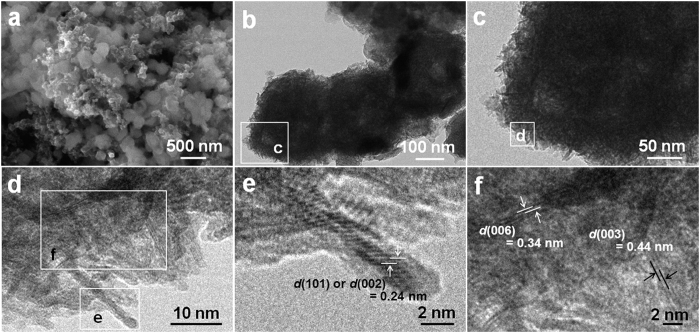
FESEM (**a**), TEM (**b-d**) and HRTEM (**e, f**) images of the hollow hetero-Ni-Co-S NBs electrode after the 100th CV cycle.

Besides the composition change of the hetero-Ni-Co-S NBs with cycling, as discussed above, the specific micro-architecture may also exhibit drastic alternation. To confirm the hypothesis, the electroactive material after cycling up to the 100^th^ cycle was flaked off from Ni foam collector for following FESEM, TEM and HRTEM evaluation. Figure 7a shows the charactistic FESEM image of the hetero-Ni-Co-S NBs after CV cycle. Clearly, numerous cube-like particles, even some with broken shell still can be distinguished. To further figure out its micro-structures more clearly, TEM measurement was carried out, and corresponding TEM images are demonstrated in Fig. 7b–d. Mesoporous fuzzy NBs unexpectedly appear, however, these unique architectures are evidently constructed with lots of nano-whiskers of ~2 nm in diameter with various stretching directions. HRTEM analysis (Fig. 7e,f) further describes the detailed geometrical structure of these nanoscaled whiskers. In Fig. 7e, the visible lattice fringes with interplanar spacing of 0.24 nm should be corresponding to the (002) plane of hexagonal phase NiOOH (JCPDS card, #06-0075) or (101) plane of rhombohedral CoOOH (JCPDS card, #07-0169). And the lattice spacing of 0.34 nm corresponds to the (006) plane of the NiOOH sample, and 0.44 nm for the (003) crystalline plane of CoOOH, as evident in Fig. 7f.

**Figure 8:**
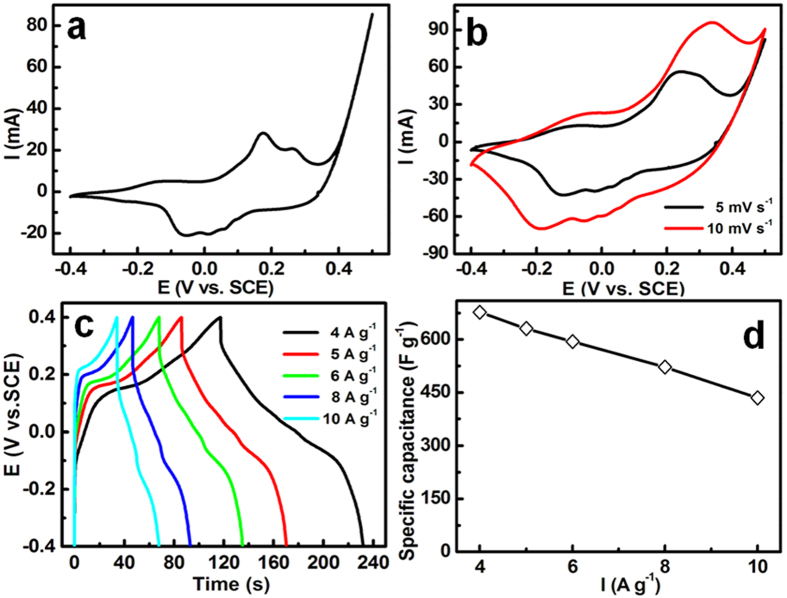
CV curves at scanning rates (a, 2 mV s^–1^; b, 5 and 10 mV s^–1^), CP plots at current densities from 4 to 10 A g^–1^ (**c**), and the SC as a function of current rates (**d**) for the hollow hetero-Ni-Co-S NBs electrode after the 100th CV cycle.

Such unique mesoporous fuzzy NBs architecture, as an advanced electroactive material for ECs, is anticipated to manifest significantly enhanced electrochemical properties with highly electrochemical accessibility and fast diffusion rates. A three-electrode system was next applied to systematically assess electrochemical properties of the electrode. Typical CV curves of the unique NBs recorded between –0.4 and 0.5 V (*vs.* SCE) at various sweep rates are shown in Fig. 8a,b. Notably, multiple redox peaks mainly distinctly appear at the potential range from –0.2 to 0.4 V, which are very similar to those who are related to the Faradaic redox couples of the Ni(III)/Ni(II) and Co(III)/Co(II), as reported before^8^,^26^−^28^. Another intensive peak above 0.4 V is designed as the oxygen evolution process. To further quality the SCs of the electrode, chronopotentiometry (CP) test was carried out at various mass-normalized current densities from 4 to 10 A g^−1^ with a stable electrochemical window of 0.8 V, and typical charge-discharge curves with a upper voltage of 0.4 V (*vs.* SCE) are illustrated in Fig. 8c. Nonlinear CP plots reveals typical Faradaic redox reactions occurring at the electrode/electrolyte sur-/interfaces, further verifying its pseudo-capacitive nature, which is in good line with the CV analysis (Fig. 8a,b). Encouragingly, the unique electrode with high loading of 7 mg cm^−2^ delivers superior pseudo-capacitance of ~677, ~631, ~594, ~522 and ~435 F g^−1^ at the large current densities of 4, 5, 6, 8 and 10 A g^−1^, respectively, which is better than single-phase sulfides (hollow Co_3_S_4_ nanospheres^17^, ~345 F g^−1^ at 5 A g^−1^; CoS nanowires^28^, 508 F g^−1^ at 2.5 mA cm^−2^; amorphous CoS^8^, 475 F g^−1^ at 5 mA cm^−2^; NiS nanoflakes^29^, 664 F g^−1^ at 4 A g^−1^), Se-doped sulfide (CuSeS_2_, 22 F g^−1^ at 4 A g^−1^)^30^, and some mixed STMs including Ni_3_S_2_@NiS (436 F g^−1^ at 0.5 A g^−1^)^26^, Ni_3_S_2_@Co_9_S_8_ (600 F g^−1^ at 0.5 A g^−1^)^26^, and so on. This observation in Fig. 8d suggests that ~64% of discharge capacitance can be maintained when the current is up to a large rate of 10 A g^−1^.

**Figure 9:**
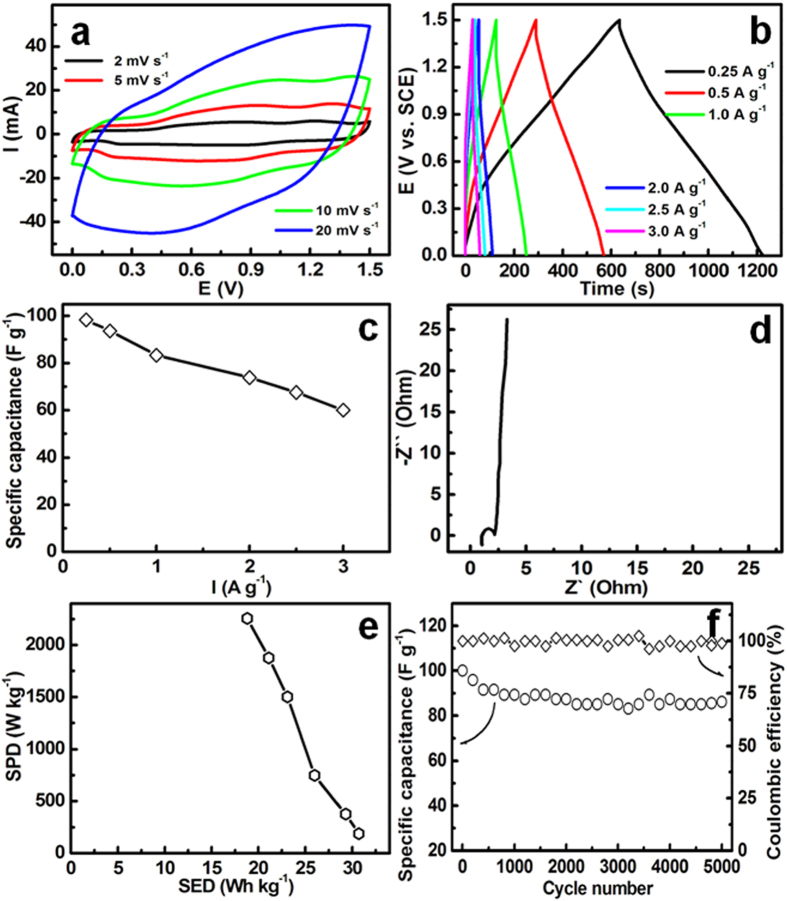
Electrochemical properties of the as-fabricated AC//hetero-Ni-Co-S asymmetric device: CV curves (**a**); CP profiles (**b**) at various current rates ranged from 0.25 to 3.0 A g^–1^, SC as a function of current density (**c**), EIS data (**d**), Ragone plot (**e**), and cycling performance and corresponding CE plot (**f**).

To further highlight its potential utilization as positive electrode for ECs, an asymmetric device with 6 M KOH as electrolyte was further fabricated by using activated carbon (AC) as negative electrode (see Supporting Information, Fig. S2). Typical electrochemical current-potential response of the aqueous asymmetric capacitor is presented in Fig. 9a. Strikingly, well-defined mirror shape with respect to the zero-current baseline, and rapid current response on potential reversals can be observed at all the scanning rates from 2 to 20 mV s^−1^, suggesting its attractive supercapacitance. One should note that the upper voltage limit of these CV curves are extended to 1.5 V, larger than those for AC//Co_3_O_4_-rGO (1.45 V)^31^, AC//RuO_2_-TiO_2_ (1.4 V)^32^, AC//CoAl double hydroxide (1.2 V)^33^, activated graphene (AG)//NiCo_2_O_4_/Cu-based (1.4 V)^34^ cells, which is greatly beneficial to the improvement in SED. Galvanostatic CP characteristics of the asymmetric device in the voltage range from 0.0 to 1.5 V were collected as a function of charge-discharge current in Fig. 9b. The nearly linear voltage variation can be seen during the electrochemical charge-discharge processes, further confirming the excellent supercapacitive behaviours of the asymmetric device. Corresponding SCs are calculated with the CP data (Fig. 9b), and recorded in Fig. 9c. Appealingly, the cell gives large charge-storage capacitances of ~99, ~94, ~84, ~74, ~68 and ~60 F g^−1^ at the current densities of 0.25, 0.5, 1.0, 2.0, 2.5 and 3.0 A g^−1^, respectively. The electrochemical impedance spectroscopy (EIS) Nyquist plot (Fig. 9d) of the asymmetric device represents good electronic conductivity (~0.92 Ohm) at a high-frequency range, revealing its small cell resistance^27^. Additionally, the small diameter of the semicircle in high-medium frequency range means small charge-transfer resistance (~1.1 Ohm) of the asymmetric device. And the linear region in low frequency leans towards imaginary axis, showing good capacitive behaviour of the asymmetric device. Figure 9e depicts the Ragone plot of the AC//hetero-Ni-Co-S asymmetric cell. Strikingly, the asymmetric device is capable of delivering a SED as large as ~31 Wh kg^−1^ based on the total weight of the electroactive materials, which is larger than other asymmtric systems such as AC//Ni_3_S_2_-CNs (~19.8 Wh kg^−1^)^13^, AC//NiO (~26.1 Wh kg^−1^)^35^, AC//Co_3_O_4_-rGO (~13.4 Wh kg^−1^)^31^, AC*//*CoAl double hydroxide (16.9 Wh kg^−1^)^33^, AC//RuO_2_-TiO_2_ (5.7 Wh kg^−1^)^32^, AC//Co(OH)_2_-USY^36^, AG//NiCo_2_O_4_/Cu-based (12.6 Wh kg^−1^)^34^, AG//NiCo_2_O_4_-MnO_2_ (9.4 Wh kg^−1^)^37^, AC//Ni-Co oxide (12 Wh kg^−1^)^38^, MnFe_2_O_4_//LiMn_2_O_4_ (5.5 Wh kg^−1^)^39^, *etc*. Furthermore, the SED is still delivered as ~18.8 Wh kg^−1^ by the asymmetric device even at a high rate with a specific power density (SPD) of 2256 W kg^−1^. The long-term cycling performance is investigated at a large constant current density of 3 A g^−1^ over 5000 cycles, as observed in Fig. 9f. The capacitance retention of ~86% suggests good electrochemical stability of the device for practical applications. Moreover, the electrochemical Coulombic efficiency (EC) of the device maintains as high as ~100% over continuous cycles.

In conclusion, a simple yet efficient self-sacrifice template synthetic platform was elegantly developed here to fabricate hollow hetero-Ni-Co-S NBs thorough chemical sulfidization of Ni_x_Co_y_CO_3_ NCs *via* effective anion-exchange process. The as-synthesized hollow hetero-Ni-Co-S NBs electrode itself exhibited no any electrochemical performance in KOH for ECs, while attractive Faradaic pesudo-capacitance was visually observed after contineously 100 scanning cycles. New insights into the underlying charge-storage mechanism of the hetero-Ni-Co-S NBs in KOH were first proposed that the electrochemically induced phase transformation gradually took place during CV sweeping from the hetero-Ni-Co-S to bi-component-active NiOOH and CoOOH, which were the intrinsic electroactive phases for the excellent Faradaic pseudo-capacitance of ~677 F g^−1^ at 4 A g^−1^ for the hollow hetero-Ni-Co-S electrode with high loading of 7 mg cm^−2^ after cycling. Furthermore, the AC//hetero-Ni-Co-S asymmetric device presented an enlarged electrochemical window of 1.5 V, rendering a maximum energy density of ~31 Wh kg^−1^. We strongly believed that our hollow design strategy, new findings and understandings will hold great promise for enriching synthetic methodologies, and electrochemistry of low-cost complex sulfides for next-generation advanced ECs.

## Methods

### The synthesis of the hetero-Ni-Co-S NBs

All the chemicals were analytic-grade reagents, and used without further purification. Typically, 0.5 mmol of Ni(CH_3_COO)_2_·4 H_2_O (Ni(AC)_2_) and 1 mmol of Co(CH_3_COO)_2_·4 H_2_O (Co(AC)_2_) was dissolved into 40 mL of ethylene glycol (EG) to form a transparent solution. Then, 30 mmol of NH_4_HCO_3_ was added into the solution under stirring for half an hour. The solution was transferred to a Teflon-lined stainless steel autoclave (50 mL), and kept at 200 °C in an electric oven for 20 h. After cooling to room temperature (RT) naturally, the light pink precipitate (denoted as Ni_x_Co_y_CO_3_) was separated by centrifugation, washed with de-ionized (DI) water and ethanol, and then dried at 80 °C.

Next, 0.6 g of Na_2_S·9 H_2_O was dissolved in 40 mL of DI water. Then, 0.2 g of the as-fabricated Ni_x_Co_y_CO_3_ was added into the above solution under stirring for 2 h. Afterwards, the mixture was transferred into a Teflon-lined stainless steel autoclave (50 mL), and kept at 120 °C for 4 h. Accordingly, black Ni-Co-S NBs were prepared. For comparison purpose, other hydrothermal durations (such as, 0.5 and 3 h) were also applied instead, and the resulted products were designed as Ni-Co-S-0.5 (0.5 h) and Ni-Co-S-3 (3 h), respectively.

### Materials Characterization

The samples were examined by powder X-ray diffraction (XRD) (Bruker, D8-Advance XRD, Germany) by using a Cu K*a* source (λ = 0.154056 nm) at a scanning speed of 2° min^−1^ over a 2*θ* range of 10–80°. The morphologies and structures were observed with field-emission scanning electron microscopy (FESEM, JEOL-6300 F, 15 kV), transmission electron microscope (TEM), high-resolution transmission electron microscope (HRTEM), and selected area electron diffraction (SAED) (JEOL JEM 2100 system operating at 200 kV). X-ray photoelectron spectroscopy (XPS) measurements were carried out on a PHi5000 X-ray photoelectron spectrometer with an Al K*a* excitation source (1486.6 eV), the spectra were fitted well with the XPSPEAK41 software. The element analysis was performed by X-Ray Fluorescene Spectrometer (XRFS, ARL Advant’X 3600).

### Electrochemical Measurementsh

The working electrode was fabricated with electroactive hetero-Ni-Co-S NBs, conductive acetylene black (AB, Super-P-Li) and polytetrafluoroethylene (PTFE) with a weight ratio of 7:2:1. A small amount of DI water was added to make more homogeneous mixture, which was then pressed onto a nickel foam (1 cm^2^) at a pressure of 15 MPa. The typical loading of the Ni-Co-S NBs is 7 mg cm^−2^. Electrochemical evaluation was carried out in three-electrode systems with platinum plate (1 cm^2^) and saturated calomel electrode (SCE) as the counter and reference electrodes, respectively. The electrolyte was 6 M KOH here. Furthermore, an asymmetric configure was constructed by using the Ni-Co-S NBs, which stabilized after cycling, and activated carbon (AC) as positive and negative electrodes, respectively, face to face in 6 M KOH electrolyte. The mass ratio of the AC to Ni-Co-S NBs is 14:5.

Electrochemical properties were evaluated by cyclic voltammetry (CV), chronopotentiometry (CP) and electrochemical impedance spectroscopy (EIS) measurements with an IVIUM electrochemical workstation (the Netherlands). The cycling performance was carried out with a CT2001D tester (Wuhan, China). The SCs of the electrode or asymmetric device were calculated from the CP plots according to the following equation:


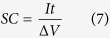


where *I, t* and Δ*V* denotes the discharging current density (A g^−1^), the discharging time (s) and the discharging potential range (V) of the electrode or asymmetric EC, respectively. Of note, the *I* was based on the two electrodes for the case of asymmetric capacitor. And the *SED* and *SPD* of the asymmetric device in 6 M KOH can be calculated by using the following equation:


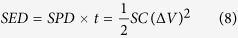


where *SC* and Δ*V* are the capacitance and working potential voltage of the asymmetric supercapacitor.

## Additional Information

**How to cite this article**: Hua, H. *et al*. Self-sacrifice Template Formation of Hollow Hetero-Ni_7_S_6_/Co_3_S_4_ Nanoboxes with Intriguing Pseudo-capacitance for High-performance Electrochemical Capacitors. *Sci. Rep.* **6**, 20973; doi: 10.1038/srep20973 (2016).

## Supplementary Material

Supplementary Information
