# Supplementary material for: Self-sacrifice Template Formation of Hollow Hetero-Ni7S6/Co3S4 Nanoboxes with Intriguing Pseudo-capacitance for High-performance Electrochemical Capacitors
Source: Sci Rep. 2016 Feb 11;6:20973. doi: 10.1038/srep20973 (PMC4750087; doi:10.1038/srep20973)
Supplement: Supplementary Information [file srep20973-s1.pdf]

Supporting Information

Self-sacrifice Template Formation of Hollow Hetero-Ni_7S_6@Co_3S_4 Nanoboxes with Intriguing Pseudo-capacitance for High-performance Electrochemical Capacitors

Hui Hua [a], *Sijia Liu* [a], *Zhiyi Chen* [a], *Ruiqi Bao* [a], *Yaoyao Shi* [a], *Linrui Hou* [a]*, *Gang Pang* [a], *Kwun Nam Hui* [b]*, *Xiaogang Zhang* [c] *& Changzhou Yuan* [a]*

[*] H. Hua, S. J. Liu, Z. Y. Chen, R. Q. Bao, Y. Y. Shi, *Prof.* L. R. Hou, *Dr.* G. Pang, *Prof.* C. Z. Yuan

[a] School of Materials Science & Engineering, Anhui University of Technology, Maanshan, 243002, P.R. China Email: houlr619@163.com; ayuancz@163.com

Prof. K. N. Hui

[b] Institute of Applied Physics and Materials Engineering, Faculty of Science and Technology, University of Macau, Macau Email: bizhui@umac.mo

Prof. X. G. Zhang

[c] College of Material Science & Engineering, Nanjing University of Aeronautics and Astronautics, Nanjing, 210016, P.R. China

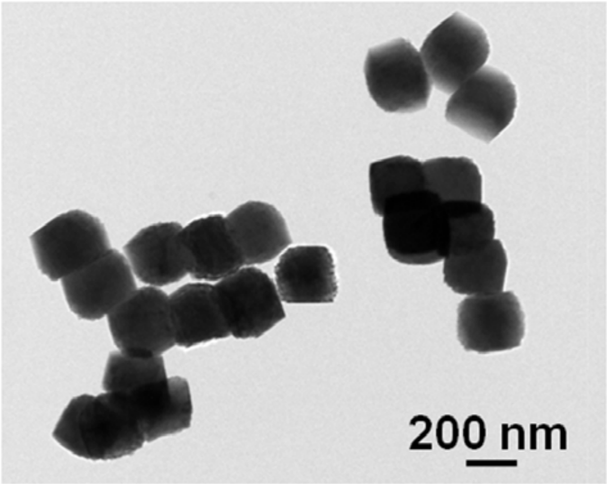

Figure S1. TEM image of the as-fabricated $Ni_xCo_yCO_3$ solid solution

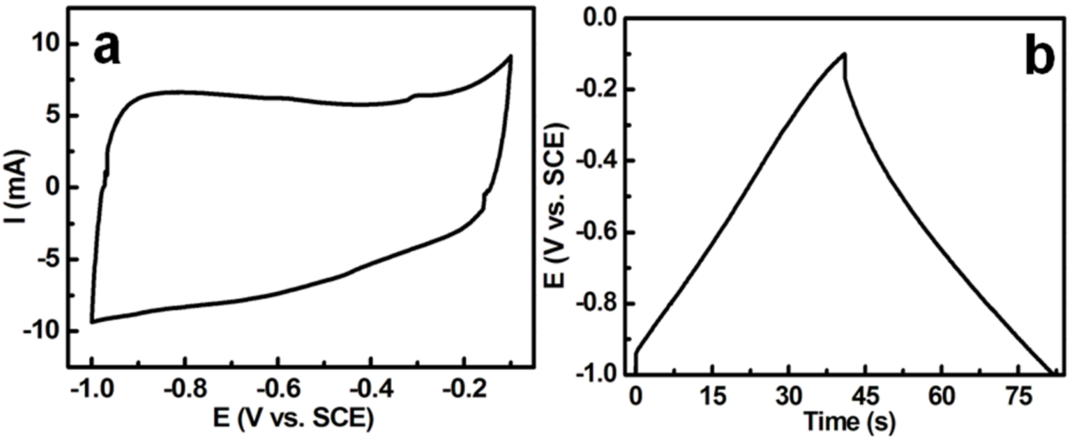

Figure S2. CV curve (a, 5 mV s^{-1}) and CP plot (b, 5 A g^{-1}) of the AC electrode

As observed in **Figure S2a**, the CV curve is nealy rectangular-like with positive sweeps nearly mirror-image symmetric to their corresponding counterparts on the negative sweeps with respect to the zero-current line, strongly demonstrating the typical electric double-layered capacitance of the AC in 6 M KOH. Linear charge-dischage plot is apparently presented in **Figure S2b**, revealing its desirable electrochemical behavior. Attractively, the unique AC exhibits remarkable specific capacitance of ~227 F g^{-1} at large current density of 5 A g^{-1}.
